# Glycyrrhizin inhibits LPS-induced inflammatory responses in goat ruminal epithelial cells in vitro

**DOI:** 10.1186/s12860-023-00489-y

**Published:** 2023-09-19

**Authors:** Junfeng Liu, Bei Ma, Guang Hao, DuoDuo Su, Tianyang Wang, Ze Ding, Xuefeng Guo

**Affiliations:** 1 College of Animal Science and Technology, Tarim University, Alar, 843300 Xinjiang PR China; 2 Department of Clinical Veterinary Medicine, College of Veterinary Medicine, Huazhong Agricultural University, Wuhan, 430070 Hubei PR China; 3 Ordos Supply and Marketing Cooperative Logistics Service Centre, Ordos, 017000 Inner Mongolia PR China

**Keywords:** SARS, GREC, Glycyrrhizin, Inflammatory mediators, mRNA expression

## Abstract

Inflammation plays a crucial role in the progression of Subacute Ruminal Acidosis (SARA). The experiment was designed to investigate anti-inflammatory effects of glycyrrhizin on goats ruminal epithelial cells (GREC) which were induced SARA by Lipopolysaccharide (LPS) in vitro. The GREC were induced SARA by adding LPS at the concentration of 5 μm and glycyrrhizin was added at different concentration of 0, 60, 90, 120, 150 μm. The structural integrity of LPS-induced GREC with the treatment of glycyrrhizin were observed by electron microscope; The levels of inflammatory factors TNF-α, IL-1β, IL-6, IL-8 and IL-12 were measured by ELISA; The number of Zo-1 and Occludin were measured, the expression of tight junction protein Occludin were measured by Western blot, and the mRNA expression of NF-κB, TNF-α, IL-1β, IL-6, IL-8 and IL-12 were measured in vitro. The results showed that higher concentration treatment of glycyrrhizin led to better morphology in LPS-induced GREC. Glycyrrhizin inhibited the growth of inflammatory factors TNF-α, IL-1β, IL-6, IL-8 and IL-12 in a dose-dependent manner. The number of ZO-1 and Occludin increased with the increase of adding of glycyrrhizin. Western blot analysis showed that the expression of tight junction protein Occludin in LPS-induced GREC increased with the adding of glycyrrhizin in a dose-dependent manner. Furthermore, the mRNA expression of NF-κB, TNF-α, IL-1β, IL-6, IL-8 and IL-12 decreased significantly with the increase treatment of glycyrrhizin. Glycyrrhizin significantly inhibits LPS-induced inflammatory mediators in GREC and the effects are better with the increase treatment of glycyrrhizin in vitro.

**Supplementary Information:**

The online version contains supplementary material available at 10.1186/s12860-023-00489-y.

## Introduction

With the growing intensity of feeding and the increasing demand for animal products, SARA has become one of the most harmful and common diseases in ruminants breeding [1]. Researches have showed that the incidence of SARA in Italy, Ireland, Netherlands and the United States was 33%, 13.8%, 11%, 19-20.1% respectively [2–5]. Yamamoto et al. [6]. Reported that when fattening cattle suffered from SARA, the rumen would be in the low acid environment for a long time, causing ruminal mucosa shedding. SARA has been reported to increase the death rate of ruminal epithelial cells and impressively damage barrier function of rumen epithelium [7]. The content of free LPS in the rumen increases sharply under the condition of SARA. LPS is the main toxin factor of *Gram negative bacteria*, when the host was infected by *Gram negative bacteria*, the monocytes and macrophages in the host could rapidly recognize LPS and thus stimulated the release of inflammatory factors such as TNF-α, IL-1β and IL-6 [8]. Ruminal epithelial cells play an important role in defensing against bacteria, but when the ruminants were under the condition of SARA, the barrier function of rumen epithelium was injured, thus LPS and inflammatory mediators could enter the blood from rumen epithelium causing systemic inflammatory reaction and infection [9]. The mechanism of LPS-induced systemic inflammatory response and infection is as follows: The LPS leads to the activation of TLR4 signaling pathway, causing the release of inflammatory cytokines. Meanwhile, When NF-κB, which has certain biological functions in cell survival, inflammation, congenital and adaptive immunity [10], was activated by LPS, it would be activated and the inflammatory mediators such as TNF-α, IL-1β would be released. A LPS-induced SARA model was established in vitro by our experiment using goats epithelial cells previously and the optimal inflammatory concentration of LPS was 5 μm. Glycyrrhizin, isolated from licorice, has been reported to have anti-oxidative [11, 12], anti-inflammatory [13, 14], antiviral such as hepatoprotection and detoxification [15, 16], and improve immunity [17] effects. In animal production, some kinds of diseases can be prevented and the meat quality of livestock can be improved by using glycyrrhizin properly [18]. However, there was few researches investigating on effects of glycyrrhizin on LPS-induced GREC. In this experiment, LPS-induced GREC were made, and different concentration of glycyrrhizin was added to investigate effects of glycyrrhizin on inflammatory mediators in vitro, the purpose of the experiment was to reveal the mechanism of glycyrrhizin inhibit on LPS-induced GREC and to provide scientific evidence for using glycyrrhizin to prevent and treat ruminants under SARA.

## Materials and methods

### Reagents

Glycyrrhizin (purity > 99%) was purchased from Shanghai Yuanye Biotechnology Co., Ltd (China). LPS was purchased from Sigma (St. Louis, MO, USA). ELISA kits for TNF-α, IL-1β, IL-6, IL-8 and IL-12 were purchased from Nanjing Institute of Bioengineering (China). BCA protein quantitative kit and SYBR Green PCR kit were purchased from Smer Fell Science and Technology Co., Ltd. (Shanghai, China). Reverse transcription kit was purchased from Ferments. RIPA, Acrylamide(29: 1), Tris-HCl pH = 8.8 electrophoretic buffer, Tris-HCl pH = 6.8 electrophoretic buffer, SDS, TEMED, PBS phosphate buffer and DEPC were purchased from JRDUN Biotechnology (Shanghai) Co., Ltd. Alexa Fluor 488 Labeled goat anti-rabbit IgG (H + L), DAPI, Goat anti-rabbit-HRP secondary sera, Donkey anti-goat-HRP secondary sera and Goat anti-mouse-HRP secondary sera were purchased from Biyuntian co., Ltd. Occludin(rabbit)and ZO-1 (rabbit) were purchased from Bioss. RMPI-1640 was purchased from Hyclone.

### Cell grouping and processing

GREC were acquired from two Boer goats (6 weeks age). Two Boer goats were provided by the Experimental Animal Center of Huazhong Agricultural University (Wuhan, China). Goats were breed in separate cages (24 ± 1 °C, 65% relative humidity) and were free to ingest foods and water. LPS-induced GREC were formulated by adding 5 μm LPS into GREC primary cell culture for 2 h, after inducing, the cells were divided into five groups with the treatment of glycyrrhizin of 0, 60, 90, 120 and 150 μm for 24 h, then cells or the supernatant was collected for further analysis.

### Detection of structural integrity of LPS-induced GREC

The fresh LPS-induced GREC with the treatment of glycyrrhizin were washed by PBS for one time, fixed by glutaraldehyde, osmic acid, each fixing followed washing by PBS for three times, dehydrated with different gradients of ethanol 30%, 50%, 70%, 90%, 95% and 100%, each time lasted for 10–15 min, replaced with propylene epoxide for two times, each time last for 10 min, permeated by the ratio of propylene oxide to epon resinand 2:1 and 1:2 for 2 h respectively, whole epon resinand for the night, polymerizates at 37 ℃ for 12 h, 45 ℃ for 24 h and 60 ℃ for 48 h, and finally, the samples were made into cell slice and observed by Transmission Electron Microscope (JEM-2100 F JEOL).

### Autophagy examination

First fixed with 2.5% glutaraldehyde for 2 h. Fix with 2% osmium acid for 2 h. Dehydrate with 50%, 70%, 80% and 90% ethanol gradients for 15 min each, and then dehydrate with 100% ethanol 3 times for 20 min each. Replace with acetone 2 times for 15 min each. Impregnate with acetone: embedding agent = 1:2 for 4 h; impregnate with pure embedding agent 2 times for 4 h each. Place the samples in the embedding plate containing pure embedding agent. The plates were polymerized at 65℃ for more than 48 h each. The embedding head was trimmed into a trapezoidal shape and the surface area of the sample was less than 0.2 mm × 0.2 mm. Slicer Sects. 50–70 nm. 3% uranyl acetate-lead citrate double staining. Observe the formation of autophagic structures, mainly including the morphology of autophagosomes and autophagic lysosomes.

### Inflammatory mediators assay

The LPS-induced GREC and the treatment of glycyrrhizin mentioned above were centrifuged at 3000 r/min for 20 min and the supernatant was collected. The level of TNF-α, IL-1β, IL-6, IL-8 and IL-12 were measured using ELISA kits according to the manufacture’s instruction (Biolegend, Camino Santa Fe, CA, USA).

### The number of Occludin and ZO-1

The LPS-induced GREC (5 × 10^5^ cells/well) were seeded in 24 well plates. Glycyrrhizin of different concentration was subsequently treated in culture medium for 24 h, cells were washed with PBS for three times, each time last for three minutes. The wells were fixed by formaldehyde, permeabilized by Triton X-100, blocked by BSA for 1 h and incubated with primary antibody and secondary antibody, each process with washing with PBS for three times, and finally, the wells were sealed and taken photos by fluorescence microscope.

### Western blot analysis

The LPS-induced GREC with the treatment of adding glycyrrhizin were disrupted sufficiently at 4 °C, centrifuged at 12 000 g for 10 min and the supernatant was collected. Total proteins were extracted with the protein extraction reagent and the concentration was measured with the BCA protein reagent. The protein was separated by SDS/PAGE and electrophoretic ally transferred onto a semi-dry rotating membrane. The membrane was blocked in NaCl/Tris containing 5% nonfat dry milk at room temperature for 1 h, and incubated with a primary antibody at 4 ℃ for 12 h and then incubated with a secondary antibody at 37 ℃ for 1 h. Finally, the blots were developed with the ECL Plus Western Blotting Detection System (GE Healthcare, Chalfont St Giles, UK).

### Primers design and synthesis

According to the mRNA sequences of NF-κB, TNF-α, IL-1β, IL-6, IL-8 and IL-12 of goats in GenBank, the upstream and downstream primers of mRNA were designed by Primer 5.0 [19, 20]. Primers were synthesized by JRDUN Biotechnology (Shanghai) Co., Ltd., as shown in (Table 1).

**Table 1:** The primer sequence of targeted genes

Gene	Primer sequences (5’-3’)	Length (bp)
NF-κB(p65) (XM_-_018043384.1)	F: GTGTAAAGAAGCGGGACTTGG R: GCGGTTGTCAAAGATGGGATG	203
TNF-α (NM_-_001286442.1)	F: CCACGTTGTAGCCAACATCAG R: AGATGAGGTAAAGCCCGTCAG	134
IL-1β (XM-013967700.2	F: CCACCTCCTCTCACAGGAAATG R: GATACCCAAGGCCACAGGAATC	100
IL-6 (NM_-_001285640.1)	F: TACCTGGACTTCCTCCAGAAC R: CGAATAGCTCTCAGGCTGAAC	245
IL-8 (XM_-_005681749.3)	F: TCTTGGCCGCTTTCCTGCTC R: TTTCGCAGTGTGGCCCACTC	162
IL-12 (NM-001285700.1)	F: GACCAAACCTCAGCCAAAG R: GACACAGATGCCCATTCAC	104
GAPDH (XM_-_005680968.3)	F: GCCCTGAGGCTCTCTTCCA	101
	R: GCGGATGTCGACGTCACA	

### RNA extraction and reverse transcription

The total RNA was extracted by using Trizol (Invitek, 1596 − 206). The first strand of cDNA was synthesized according to the instructions of Dalian Bao Biological engineering Co., Ltd and was synthesized according to the reverse transcription kit (Fermentas, #K1622) [21]. The reaction system was showed in (Table 2). The reverse transcription was carried out at 37 ℃ for 60 min, 85 ℃ for 5 min and 4 ℃ for 5 min.

**Table 2:** The system of reverse transcription reaction

Reagents	Addition
RNA-Primer Mix	12 µL
5×RT Reaction Buffer	5 µL
25 mM dNTPs	1 µL
25 U/µL RNase Inhibitor	1 µL
200 U/µL M-MLV Rtase	1 µL
Oligo(dt)18	1 µL
ddH_2_O (DNase-free)	4 µL
Total volume	25 µL

### Real-time PCR

Then the cDNA was subjected to PCR amplification after autoclaving and diluting to 10 μm with distilled water with SYBR Green PCR kits (Thermo, #K0223). The reaction system was shown in (Table 3) and response procedure was that: 95 ℃, 10 min; (95 ℃, 15 s; 60 ℃, 45 s) ×40 cycles; 95 ℃, 15 s; 60 ℃, 1 min; 95 ℃, 15 s; 60 ℃, 15 s.

**Table 3:** The system of PCR reaction

Reagents	Addition
SYBRGreen Mix	12.5 µL
Upstream primer F	0.5 µL
Downstream primer R	0.5 µL
ddH_2_O	9.5 µL
cDNA templet	2 µL
Total volume	25 µL

### The mRNA expression of NF-κB, TNF-α, IL-1β, IL-6, IL-8 and IL-12

After Real-time PCR amplification, the relative mRNA expression of NF-κB, TNF-α, IL-1β, IL-6, IL-8 and IL-12 were measured with standard mRNA curve.

### Statistical analysis

All results were expressed as the means ± SEM with SPSS 16.0. Comparisons between groups were performed with ANOVA followed by Duncan test. Differences were considered to be significant at *P* < 0.05 or *, extremely significant at *P* < 0.01 or **.

## Results

### Effect of glycyrrhizin on structural integrity of LPS-induced GREC

**Fig. 1:**
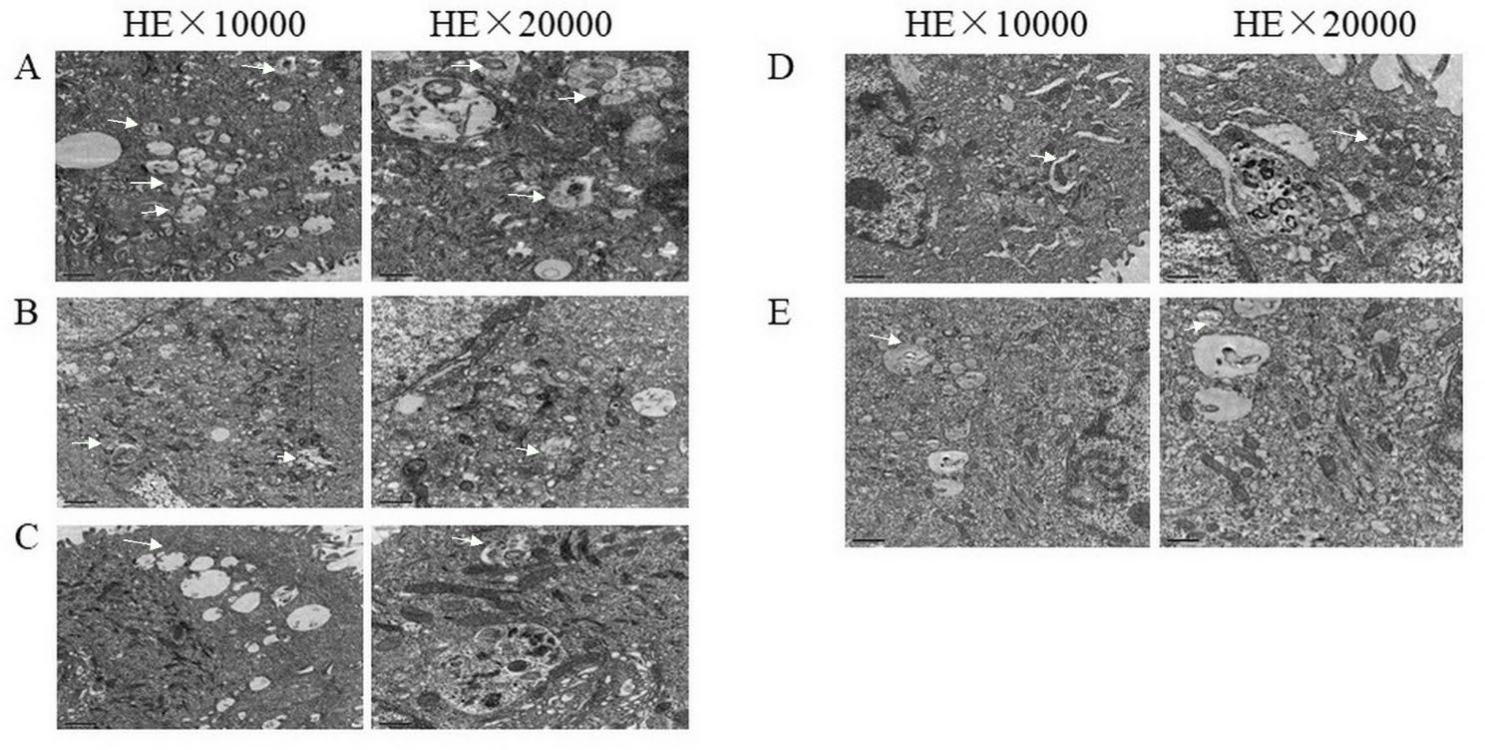
**(A-E)**. Effects of glycyrrhizin on structural integrity in LPS-induced GREC. Observation of morphological changes in different stages of autophagy using transmission electron microscopy. Figure 1 **A**, **B**, **C**, **D** and **E** was investigated at the concentration of glycyrrhizin was 0, 60, 90, 120, 150 μm respectively. The autophagy around the nucleus was the highest in picture **A**, and gradually decreased in picture **B**, **C**, **D** and **E**

Qualitative examination, LPS-induced GREC in the cytoplasm, autophagic vesicles and autophagic lysosomes were formed; including crescentic or cup-shaped, bilayer or multilayer membranes; bilayer or multilayer membranes with vesicle-like structures, etc. Monolayers with degraded cytoplasmic components. As shown (Fig. 1), the autophagy is most evident in Fig. 1A. In Fig. 1B, C, D and E, autophagy decreases in a dose-dependent manner with the addition of glycyrrhizin; and the best cell morphology of GREC is observed when the glycyrrhizin concentration is 150 μm.

### Effects of glycyrrhizin on inflammatory mediators in LPS-induced GREC

**Fig. 2:**
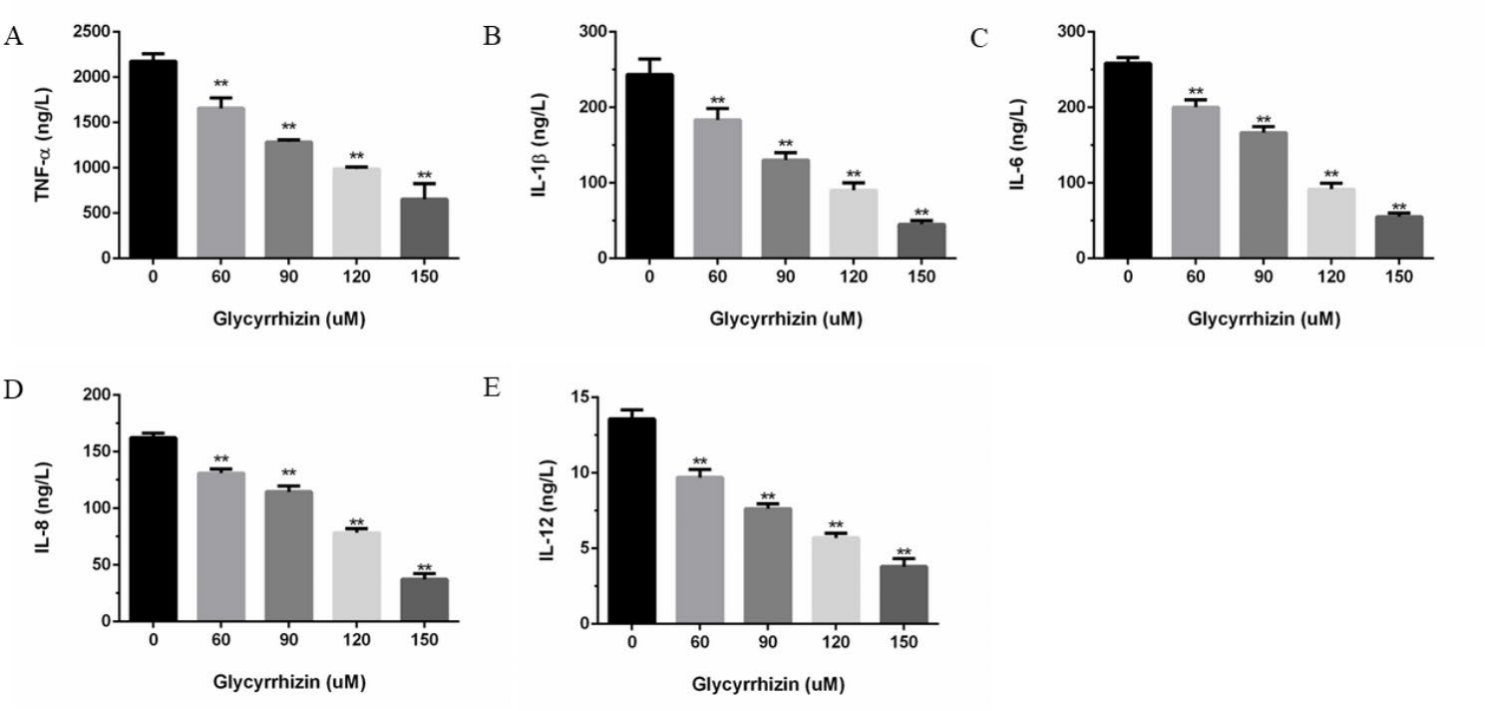
**(A-E)**. Effects of glycyrrhizin on inflammatory mediators TNF-α **(A)**, IL-1β**(B)**, IL-6**(C)**, IL-8**(D)** and IL-12**(E)** in LPS-induced GREC. The data presented were the means ± S.E.M of three independent experiments. **p < 0.01, *p < 0.05 versus LPE-induced GREC without treatment of glycyrrhizin

The effects of glycyrrhizin on LPS-induced TNF-α, IL-1β, IL-6, IL-8 and IL-12 were measured to investigate the anti-inflammatory effects of glycyrrhizin. As showed in (Fig. 2A, B, C, D, E), the production of inflammatory mediators in LPS-induced GREC without treatment of glycyrrhizin was significantly higher than those with the treatment of glycyrrhizin (*P* < 0.01) and the treatment of glycyrrhizin inhibited LPS-induced inflammatory mediators production in a dose-dependent manner.

### Effects of glycyrrhizin on the number of protein of ZO-1 and Occludin in LPS-induced GREC

**Fig. 3:**
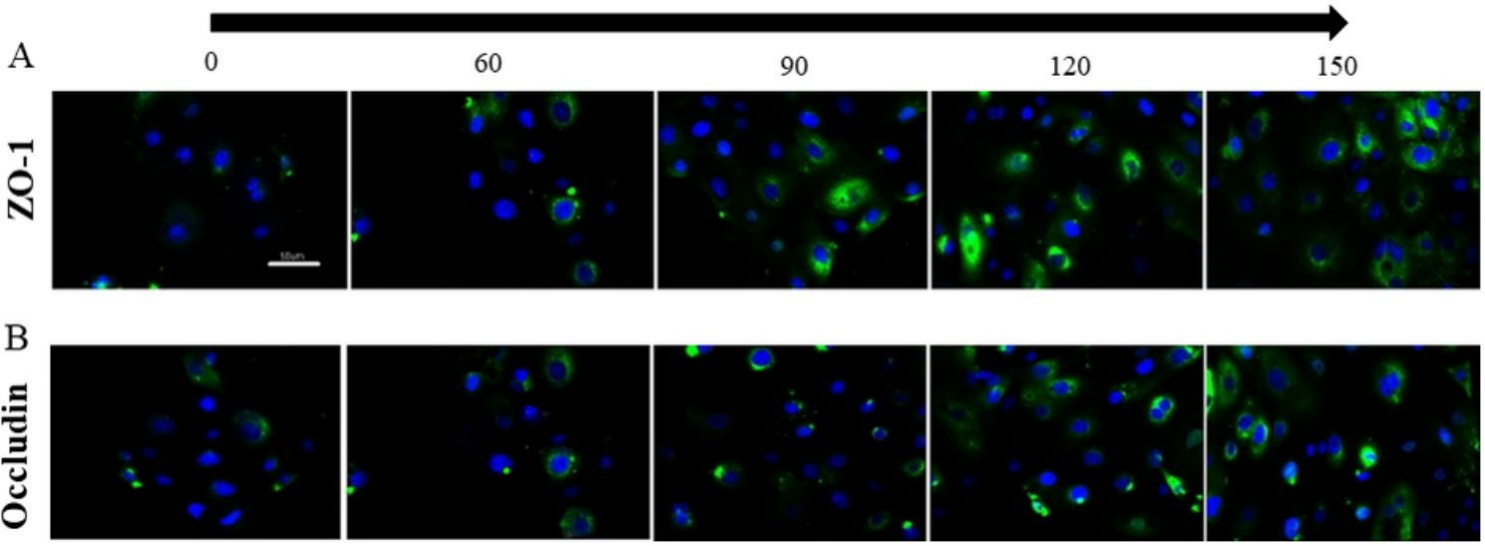
**(A-B)**. Effects of glycyrrhizin on the number of protein of ZO-1**(A)** and Occludin **(B)**. The number of the ZO-1(the blue color was nucleus and the green is ZO-1) and Occludin (the blue color was nucleus and the green is Occludin, ×400, scale = 50 μm)). From left to right the LPS-induced GREC were investigated at the adding concentration of glycyrrhizin at 0, 60, 90, 120, 150 μm. The number of ZO-1 and Occludin increased with the increase adding of glycyrrhizin

To further confirm effects of glycyrrhizin on LPS-induced GREC, the number of Occludin and ZO-1 was measured and showed in (Fig. 3A, B). With the increase of glycyrrhizin, the number of Occludin and ZO-1 significantly increased and the number of Occludin and ZO-1 was the most when the concentration of glycyrrhizin was 150 μm.

### Effects of glycyrrhizin on the expression of Occludin

**Fig. 4:**
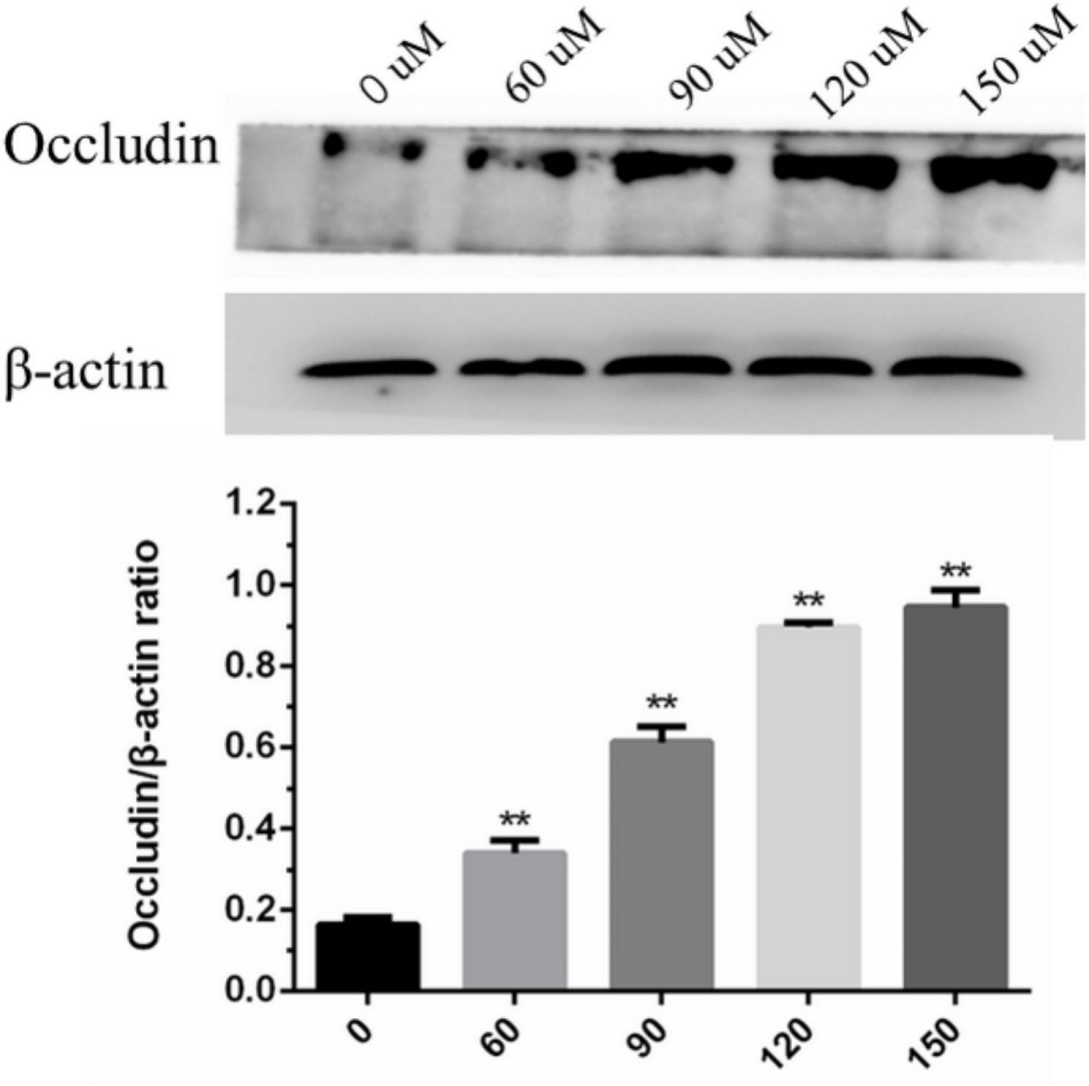
Effects of glycyrrhizin on the expression of Occludin. Apply Image Pro Plus (IPP) 6.0 software to calculate the grayscale value of the protein. β-actin was used as control. Blots were cut prior to hybridization with the antibody. Include images of all blots and all duplicate images in the supplement

The content of Occludin was shown in Fig. 4, with the increase treatment of glycyrrhizin, the content of Occludin gradually increased and the concentration reached the highest when the concentration of glycyrrhizin was 150 μm.

### Effects of glycyrrhizin on mRNA expression of NF-κB, TNF-α, IL-1β, IL-6, IL-8 and IL-12 in LPS-induced GREC

**Fig. 5:**
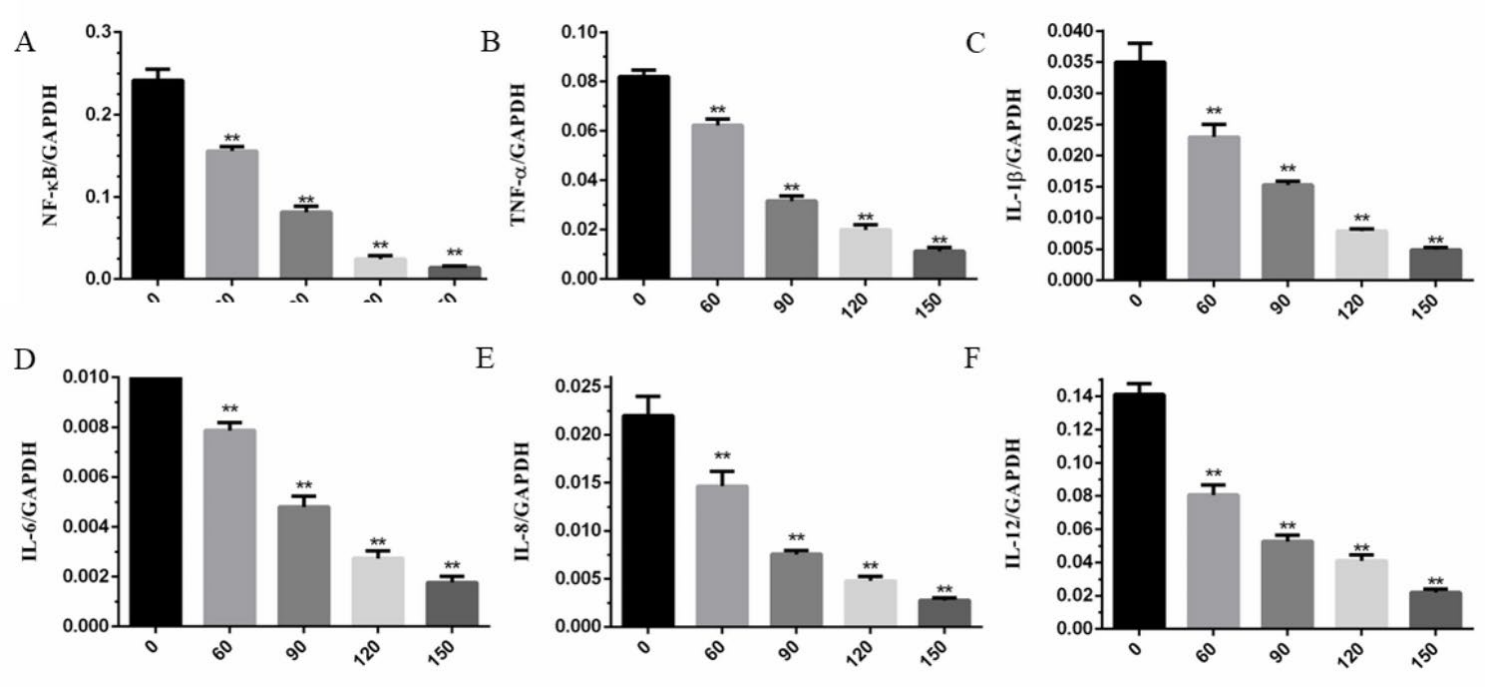
**(A-F)**. Effects of glycyrrhizin on the mRNA expression of NF-κB **(A)**, TNF-α **(B)**, IL-1β**(C)**, IL-6**(D)**, IL-8**(E)** and IL-12**(F)**. Glycyrrhizin decreased the mRNA expression of inflammatory mediators in a dose-dependent manner. The data presented were the means ± S.E.M of three independent experiments. ** p < 0.01, * p < 0.05 versus LPS-induced GREC without treatment of glycyrrhizin

As showed in Fig. 5A, B, C, D, E, F; the mRNA expression of NF-κB, TNF-α, IL-1 β, IL-6, IL-8 and IL-12 in LPS-induced GREC was extremely higher than those with the treatment of glycyrrhizin (*P* < 0.01) and glycyrrhizin inhibited the mRNA expression of these inflammatory cytokines in a dose-dependent manner. Effects of inhibition were the best when the concentration of glycyrrhizin was 150 μm.

## Discussion

There are many researches investigating on the anti-inflammatory and antiviral effects of Glycyrrhiza glabra [22], which has been used to treat disorders like endometriosis and mastitis. Glycyrrhizin is the major ingredient of Glycyrrhiza glabra. Wang et al. [23] reported that glycyrrhizin could inhibit LPS-induced inflammatory mediators in endometrial epithelial cells. Inflammation plays a crucial role in the progression of SARA. It was reported that when the ruminants were under SARA, the LPS and inflammatory mediators increased. Emmanuel et al. [24]. Reported that when the level of LPS was 5 µg/mL in rumen in vitro, the permeability of rumen increased, LPS easily penetrated the rumen wall into the blood. In this experiment, effects of glycyrrhizin on LPS-induced GREC were investigated. The integrity of cells are very important for cells to be live. In this experiment, the structural integrity of LPS-induced GREC became better with the treatment of glycyrrhizin and the structural integrity of LPS-induced GREC become well with the increase adding of glycyrrhizin, indicating that glycyrrhizin could protect and treat the cellular morphology of LPS-induced GREC.

When the body is infected by external bacteria, inflammatory cytokines that regulate and control inflammatory responses are released, including TNF-α, IL-1β, IL-6, IL-8 and IL-12. LPS induced monocyte phagocytes to release IL-1, IL-6, TNF-α and other inflammatory factors after entering the blood to participate in circulation. In this experiment, the results showed that glycyrrhizin successfully inhibited inflammatory mediators in a dose-dependent manner, which was consistent with Fu et al. [25].

The rumen epithelium from mucous layer to serosa was stratum carenum, stratum granulosum, and stratum spinosum and stratum basales. There is tight connection protein in stratum granulosum, which plays an important role in maintaining rumen barrier function [26, 27]. The tight junction protein are composed of Claudin protein, Occluding protein, ZO-1, ZOs and so on. Sun et al. [28]. reported that when the epithelial cells were under SARA, pH interacted with LPS or HIS worked on rumen epithelium and decreased the mRNA expression of tight junction protein in rumen epithelium in vitro. When the ruminal epithelial cells were induced by LPS, the tight junction protein was destroyed, the cell gap became larger. By investigating, the number of the tight junction proteins can determine whether pathological changes had occurred in rumen epithelium. In this experiment, immunofluorescence was used to investigate the distribution of Occludin and ZO-1. The number of Occludin and ZO-1 increased significantly in LPS-induced GREC with the treatment of glycyrrhizin. The results further verified that glycyrrhizin protected rumen epithelium and restored the function of cell barrier. TNF-α is an anti-tumor multi-function factor which can promote the tumor necrosis and a large amount of TNF-α can cause serious inflammatory reactions such as blood pressure increase and tissue necrosis. TNF-α is the main signal transduction molecule of LPS in organism reaction and it can inhibit the expression of Occludin largely [29–31].Thus, the content of Occludin was measured in this experiment to investigate on whether glycyrrhizin could increase the content of Occludin. Results showed that the content of Occludin in LPS-induced GREC was a little, and the content of Occludin increased with the treatment of glycyrrhizin in a dose-dependent manner. These results may indicate that glycyrrhizin could inhibit the expression of TNF-α, which needs to be further investigated.

Stimulating ruminal epithelial cells with LPS lead to the activation of NF-κB signaling pathway [32]. NF-κB signaling pathway has attracted widespread attention for its ability to regulate transcription of inflammatory mediators [33]. By activating NF-κB, LPS can increase the transcription of TNF-α, IL-1β and IL-6, inducing the expression of pro-inflammatory response mediators [34]. NF- κB is considered to be an important pathogenic factor in many acute and chronic inflammatory diseases, thus increase the efficiency of inhibiting NF-κB can effectively treat inflammatory diseases [35]. Effects of glycyrrhizin on mRNA expression of TNF-α, IL-1β, IL-6, IL-8 and IL-12 in NF-κB signaling pathway in LPS-induced GREC were investigated and the results showed that with the increase treatment of glycyrrhizin, the mRNA expression of NF-κB, TNF-α, IL-1β, IL-6, IL-8 and IL-12 decreased significantly. The results showed that glycyrrhizin inhibited the LPS-induced inflammatory mediators at the genetic level.

However, in this experiment, glycyrrhizin inhibited LPS induced cellular inflammatory damage; And its impact on cell autophagy. In the future, animal experiments will reveal the ability of glycyrrhizin to inhibit inflammation and autophagy.

### Electronic supplementary material

Below is the link to the electronic supplementary material.

Supplementary Material 1

## Data Availability

The datasets used and/or analyzed during the current study are available from the corresponding author on reasonable request.
