# Supplementary material for: Glycyrrhizin inhibits LPS-induced inflammatory responses in goat ruminal epithelial cells in vitro
Source: BMC Mol Cell Biol. 2023 Sep 19;24:28. doi: 10.1186/s12860-023-00489-y (PMC10507872; doi:10.1186/s12860-023-00489-y)
Supplement: Supplementary file 1 — Supplementary Material 1 [file 12860_2023_489_MOESM1_ESM.docx]

**Gel scans from the main figures**

The original image of the repeated experiment.

**Fig. 4**

**1: 0 uM 2: 60 uM 3: 90 uM 4: 120 uM 5: 150 uM**

**
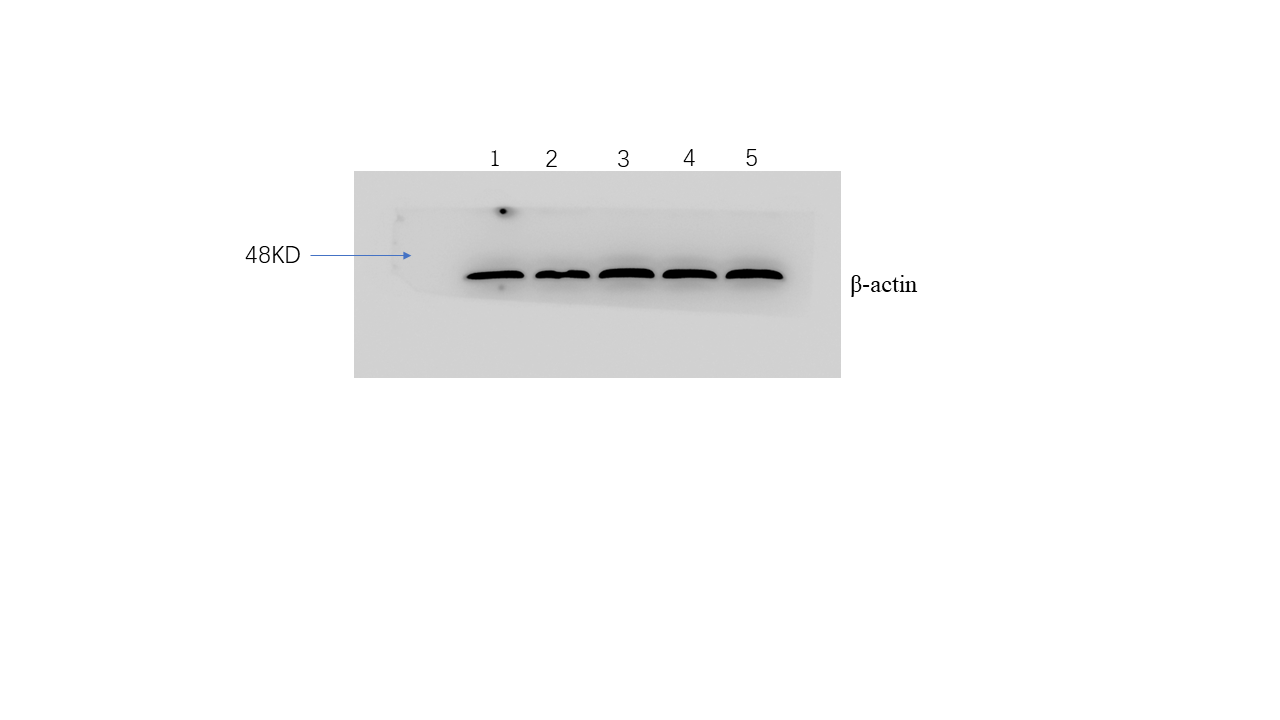
**


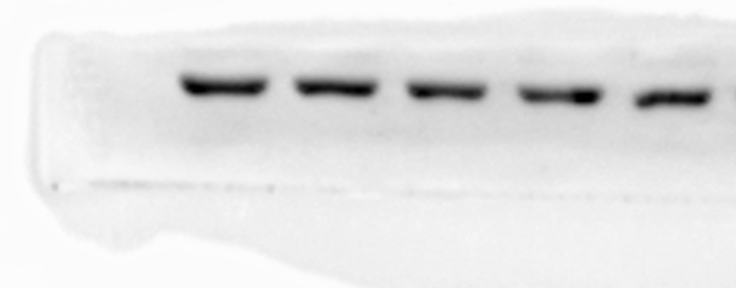


β-actin


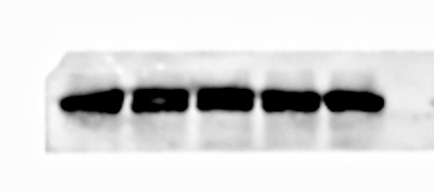

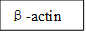


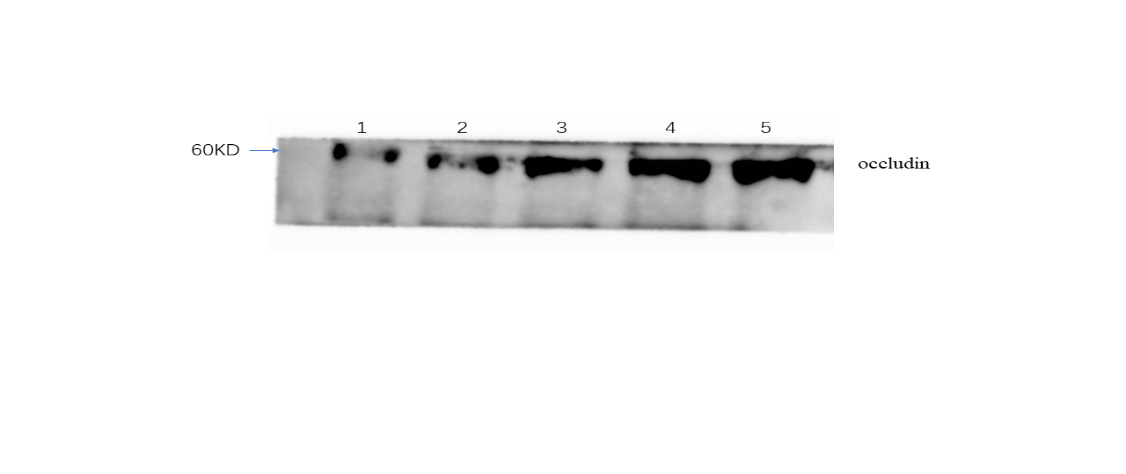


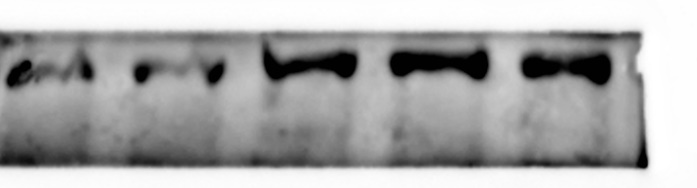
occludin


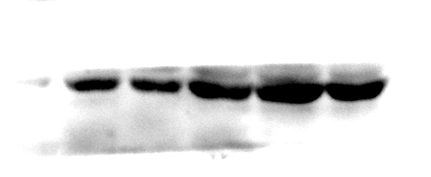
 occludin
